# Occupational cold exposure and symptoms of carpal tunnel syndrome – a population-based study

**DOI:** 10.1186/s12891-022-05555-8

**Published:** 2022-06-21

**Authors:** Albin Stjernbrandt, Per Vihlborg, Viktoria Wahlström, Jens Wahlström, Charlotte Lewis

**Affiliations:** 1 Department of Public Health and Clinical Medicine, Section of Sustainable Health, Umeå University, 901 87 Umeå, Sweden; 2 Department of Geriatrics, Faculty of Medicine and Health, Örebro University, Örebro, Sweden

**Keywords:** Carpal Tunnel Syndrome, Paresthesia, Cold Exposure, Cold Injury, Vibration, Lifting, Ergonomics, Occupational Exposure, Sweden, Occupational Health

## Abstract

**Background:**

Cold exposure is an underrecognized occupational hazard that may increase the risk of peripheral nerve entrapment. The aim of this study was to determine if self-reported occupational exposure to contact and ambient cooling was associated with symptoms of carpal tunnel syndrome (CTS).

**Methods:**

In this mainly cross-sectional study, surveys were conducted on a population-based sample of men and women between 18 and 70 years of age, living in northern Sweden. Cold exposure and presence of symptoms suggestive of CTS were subjectively reported. Associations between exposure and outcome were evaluated using logistic regression.

**Results:**

The study included 2,703 women and 2,314 men, with a median age of 60 years (interquartile range 19). Symptoms of CTS were reported by 453 (9.2%). Being highly occupationally exposed (almost always) to contact cooling of the hands was associated with reporting CTS (OR 3.20; 95% CI 1.62–6.33), as was ambient cooling (OR 2.00; 95% CI 1.03–3.88) and severe ambient cooling (OR 4.02 95% CI 2.09–7.71), after adjusting for age, gender, body mass index, current daily smoking, diabetes mellitus, joint disease, and hand-arm vibration exposure. The point estimates increased with longer daily exposure duration. For workers exposed to severe ambient cooling for more than half of their working hours, in addition to performing heavy manual handling every day, the OR for reporting CTS was 7.25 (95% CI 3.88–13.53), with a positive additive interaction effect (expressed as relative excess risk due to interaction) of 4.67.

**Conclusions:**

Self-reported occupational exposure to contact and ambient cooling was associated with symptoms suggestive of CTS. There were statistically significant positive exposure–response patterns for time spent exposed to contact and ambient cooling at work in relation to reporting symptoms of CTS. Positive additive interaction effects between cold exposure and heavy manual handling were also found. Since there was important potential uncontrolled confounding regarding repetitive wrist movements and forceful gripping, the results need to be confirmed by other studies, preferably with longitudinal design and more detailed exposure assessment.

**Supplementary Information:**

The online version contains supplementary material available at 10.1186/s12891-022-05555-8.

## Background

Carpal tunnel syndrome (CTS) is caused by compression of the median nerve passing under the transverse carpal ligament in the wrist. It is characterized by sensory and motor symptoms and signs in distribution area of the median nerve, mainly distal to the wrist. CTS is the most common entrapment neuropathy, with prevalence rates in the general population ranging from 6 to 19% depending on case definition, and with a consequent female predominance [1, 2]. Incidence rates close to 300 cases per 100,000 person years have been estimated, and CTS has been reported to constitute over 60% of work-related upper limb musculoskeletal disorders in Europe [3]. In a Swedish prevalence study, symptoms of CTS in a postal survey was reported by roughly 14%, while a neurophysiological diagnosis could be confirmed in about 4% of subjects [4]. Having CTS can affect the work ability by reducing sensory and motor function of the hand, as well as negatively affecting sleep quality through nocturnal dysesthesia. Undergoing surgical carpal tunnel release can also be followed by absence from work for two to three months among subjects with strenuous manual work [1]. Even more striking is the fact that only roughly half of surgically treated CTS patients return to their previous occupation without any changes in work tasks [5], due to incomplete relief of CTS symptoms or postoperative hyperalgesia in the carpal area. According to the previous literature, the risk of developing CTS is affected by the presence of previous fracture or local trauma, joint disease, obesity, diabetes mellitus, rheumatic diseases, endocrine disorders, and pregnancy [6–8]. In addition, several occupational risk factors have been established, including repetitive wrist movements, forceful gripping, and exposure to hand-arm vibration (HAV) [6, 9, 10].

Occupational *ambient cold exposure* can be defined as working in temperatures below 10 °C [11], which modified by windspeed and humidity imposes a general cooling effect on the entire body that can cause hypothermia during severe conditions. As a separate entity, *contact cooling* occurs when parts of the body are in contact with cold objects or liquids (e.g. gripping cold tools or submerging the hands in cold water), and this can produce a pronounced local cooling effect which increases the risk of local cold injury, but very seldomly affects the overall thermal balance [12]. The cooling effect is subsequently modified by several individual factors, including the insulating capacity of clothing (including the use of protective gloves), body composition, and physical activity level. In a study on frozen food factory workers, there was a roughly nine-fold increased odds ratio (OR) for CTS among those exposed to both contact cooling of the hands and repetitive wrist movements, compared to a mere doubling of the OR for those only exposed to repetitive work [13]. In another study on occupational risk factors for CTS, working in a cold environment was associated with a roughly fourfold increased OR for surgically treated CTS [5]. Further, in a neurophysiological outpatient clinic, a much higher frequency of CTS findings were present in the winter (51%) compared to the summer (39%), indicating a clear seasonal variation [14]. Apart from mere cold exposure, manifest local cold injury has also been reported to predispose to CTS surgery [15]. In addition, there is an abundance of literature describing peripheral sensory nerve dysfunction and injury in general from cooling, in both animal models [16, 17] and human subjects [18, 19]. However, the opposite causal mechanism has also been suggested, where peripheral nerve compression could increase the susceptibility to contact cooling [20], and predispose to local cold injury [15]. To date, there have been no previous population-based studies designed to explore the potential excess risk of CTS among cold-exposed workers, and an exposure–response function has not been demonstrated.

The primary aim of this study was to determine if self-reported occupational exposure to contact and ambient cooling was associated with symptoms of CTS. Secondary aims were to assess the presence of exposure–response patterns for occupational cold exposure, and investigate potential interaction effects between occupational cold and biomechanical exposures.

## Methods

### Study design and setting

This population-based study was part of a research project called *Cold and Health in northern Sweden*, initiated in 2015 to investigate cold-related health effects. Three surveys have been sent to the cohort. This study is mainly of cross-sectional design and based on the most recent survey, where the data was collected during the late winter months (March and April) of 2021. The study population consisted of men and women between 18 and 70 years of age at the time of recruitment, living in northern Sweden, sampled from the Swedish population register. The sampling and data collection have previously been described in detail [21].

### Description of materials

Since continuous variables were not normally distributed, data were described as median values and interquartile ranges (IQR), while categorical variables were presented as numbers and valid row percentages, unless otherwise specified. Cases with CTS symptoms were defined using two questionnaire items: *having tingling or numbness in the thumb, index and middle finger*; and *having nocturnal numbness in the hands.* Answers were given on a four-graded scale ranging from *none* to *a lot*, where *a lot* was considered a positive response. To be defined as having CTS symptoms, positive responses (*a lot*) were required on both items. All other subjects were considered to be healthy references. Occupational cold exposure was assessed using three questionnaire items: *contact cooling (handling of cold objects with a temperature at or below 0 °C with the hands)*; *ambient cooling (being exposed to cold environments such as outdoor work in the winter, work in refrigerated rooms or similar)*; and *severe ambient cooling (being exposed to cold, moisture, and wind that induces cooling despite adequate clothing).* For HAV exposure, one questionnaire item was used: *being exposed to vibration from handheld machines or tools (e.g. a drilling machine)*. Answers were given on a six-graded time scale ranging from *never* to *almost always*. Any presence of local cold injuries in the hands was also asked about. In addition, one questionnaire item was used to assess heavy manual handling, asking about the frequency of *lifting at least 15 kg per unit multiple times per day*, with answers on a five-graded time scale, ranging from *never* to *every day*. Additional independent variables used for adjusting were: *age (years)*; *gender (male/female)*; *body mass index (BMI; kg/m*^*2*^*)*; *current daily smoking (yes/no)*; *physician-diagnosed diabetes mellitus (yes/no)*; and *physician-diagnosed joint disease (yes/no)*. Occupation was reported in free-form text, and manually coded using the major and sub-major groups of the International Standard Classification of Occupations (ISCO) [22]. To enable a complementary longitudinal analysis, we also used another questionnaire item: *During work I am exposed to outdoor or cold environments,* where responses were given on a numerical rating scale (NRS) ranging from 1 *(do not agree)* to 10 *(fully agree)*, and subsequently dichotomized based on the 50^th^ percentile. Data on occupation, cold exposure using the NRS, and the presence of diabetes mellitus or joint disease were retrieved retrospectively from the initial survey performed in 2015, while all other data were collected in 2021.

### Statistical analysis

Age was categorized into four similar spans, and BMI separated by clinically used thresholds for under- and overweight [23]. Binary logistic regression was used for simple and multiple analyses. Correlation between scales was investigated using Spearman’s rank correlation coefficient. Calculations on additive interaction effects, expressed as relative excess risk due to interaction (RERI), were performed using formulas described by VanderWeele and Knol [24]. A *p* value < 0.05 was considered statistically significant. Statistical analyses were performed using IBM SPSS Statistics for Windows (Version 27, IBM Corporation, Armonk, NY, USA).

## Results

### Participants and descriptive data

In total, there were 5,208 responses to the 2021 survey, yielding a response rate of 44.4%. Due to multiple or erroneous data entries, 191 survey responses could not be used, which left 5,017 subjects available for analysis. The median (IQR) age was 60 (19) years, and 2,703 (53.9%) subjects were female. Experiencing numbness or tingling in the thumb, index and middle finger was reported by 658 (13.3%) subjects, while nocturnal numbness was reported by 888 (18.0%) subjects. Giving positive responses to both items fulfilled the case definition, as was done by 453 (9.2%) subjects. Further descriptive data are presented in Table 1, and gender-stratified data available in Additional file 1.

**Table 1:** Descriptive characteristics of the study participants

**Variable**	**Categories**	**Carpal tunnel syndrome**		**References**	
		N	%	N	%
Participants	None	453	9.2 ^a^	4,456	90.8 ^a^
Gender	Male	180	39.7	2,078	46.6
	Female	273	60.3	2,378	53.4
Age group (years)	18–31	9	2.3	207	5.7
	32–44	44	11.2	618	16.9
	45–57	136	34.6	1,115	30.6
	58–70	204	51.9	1,708	46.8
Body mass index (kg/m^2^)	BMI < 18.5	6	1.4	36	0.8
	18.5 ≤ BMI < 25	133	30.0	1,881	43.1
	25 ≤ BMI < 30	191	43.1	1,707	39.2
	BMI ≥ 30	113	25.5	736	16.9
Current daily smoking	Yes	21	4.6	165	3.7
	No	431	95.4	4,260	96.3
Geographical location	Coastal	211	46.6	2,438	54.7
	Inland	126	27.8	1,138	25.5
	Alpine	116	25.6	880	19.7
Occupational group ^b^	Service and sales workers	83	18.9	518	11.9
	Professionals	70	15.9	1,003	23.0
	Retired	61	13.9	664	15.2
	Technicians and associate professionals	44	10.0	545	12.5
	Plant and machine operator	41	9.3	241	5.5
	Clerical support workers	37	8.4	431	9.9
	Crafts workers	31	7.1	202	4.6
	Managers	18	4.1	240	5.5
	Self-employed	14	3.2	82	1.9
	Sick or parental leave	14	3.2	61	1.4
	Manual workers	13	3.0	85	1.9
	Agricultural and fishery workers	5	1.1	56	1.3
	Unemployed	5	1.1	69	1.6
	Students	3	0.7	149	3.4
	Professional militaries	0	0	18	0.4

^a^Column percentage

^b^According to the International Standard Classification of Occupations (ISCO-08)

### Cold exposure and symptoms of carpal tunnel syndrome

**Fig. 1:**
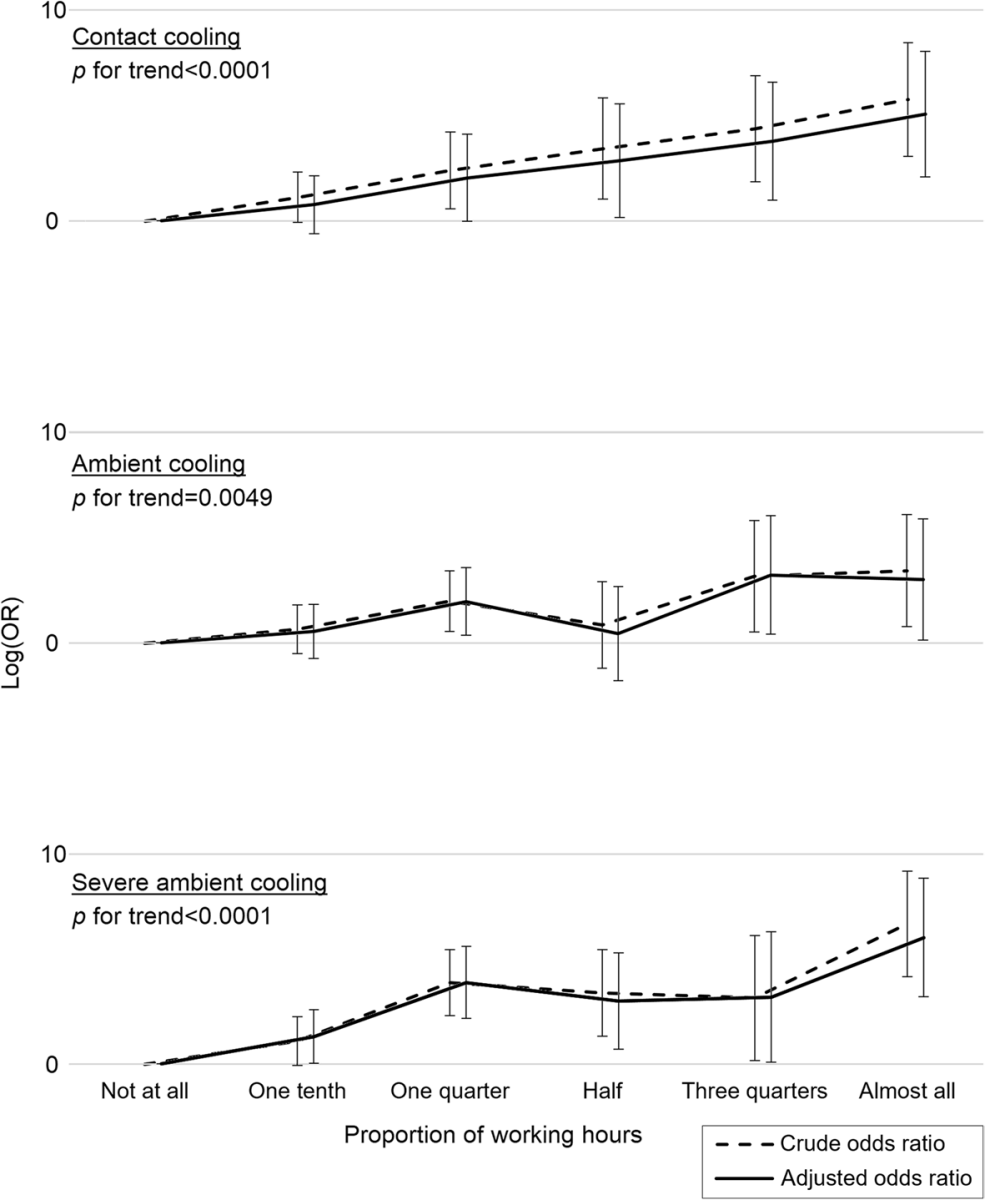
Exposure–response patterns for occupational cold exposure in relation to reporting carpal tunnel syndrome. Dashed lines represent crude odds ratios, while solid lines have been adjusted for age, gender, body mass index, current daily smoking, diabetes mellitus, joint disease, and hand-arm vibration exposure. Error bars depict ninety-five percent confidence intervals

Being highly occupationally exposed (almost always) to contact cooling of the hands was associated with reporting CTS symptoms (OR 3.20; 95% CI 1.62–6.33), as was ambient cooling (OR 2.00; 95% CI 1.03–3.88) and severe ambient cooling (OR 4.02; 95% CI 2.09–7.71), after adjusting for age, gender, BMI, current daily smoking, diabetes mellitus, joint disease, and HAV exposure (Table 2). There were positive exposure–response trends for all three exposure variables, where increasing exposure duration resulted in higher point estimates for reporting CTS symptoms. The positive linear trends were statistically significant for all three exposure variables, but not every exposure duration category (Fig. 1). Apart from adjusting for gender, the logistic regression analyses were also stratified for gender (Additional file 1), where most effects were more prominent in males. Besides cold exposure, having actually sustained a local cold injury in the hands was also associated with reporting CTS symptoms (OR 1.65; 95% CI 1.22–2.22, after adjusting for age, gender, BMI, current daily smoking, diabetes mellitus, joint disease, and HAV exposure). In a complementary longitudinal analysis, reporting high occupational cold exposure (above the 50^th^ percentile) in 2015 was also associated with reporting CTS symptoms in 2021 (OR 1.53; 95% CI 1.22–1.93), after adjusting for the same covariates as above.

**Table 2:** Logistic regression for occupational cold exposure in relation to reporting symptoms of carpal tunnel syndrome

Exposure variable	Exposure level	Carpal tunnel syndrome	References	Simple analyses	Multiple analyses ^a^
	Proportion of working hours	N (%)	N (%)	Crude OR (95% CI)	Adjusted OR (95% CI)
Contact cooling	Never	254 (63.3)	2,869 (73.9)	Reference	Reference
	One tenth	74 (18.5)	645 (16.6)	1.30 (0.99–1.70)	1.19 (0.87–1.63)
	One quarter	28 (7.0)	182 (4.7)	**1.74 (1.14–2.64)**	1.60 (0.99–2.56)
	Half	16 (4.0)	82 (2.1)	**2.20 (1.27–3.82)**	**1.92 (1.04–3.57)**
	Three quarters	15 (3.7)	62 (1.6)	**2.73 (1.53–4.87)**	**2.38 (1.26–4.52)**
	Almost always	14 (3.5)	42 (1.1)	**3.77 (2.03–6.99)**	**3.20 (1.62–6.33)**
Ambient cooling	Never	227 (56.2)	2,470 (63.6)	Reference	Reference
	One tenth	82 (20.3)	766 (19.7)	1.17 (0.89–1.52)	1.14 (0.85–1.53)
	One quarter	48 (11.9)	329 (8.5)	**1.59 (1.14–2.21)**	**1.57 (1.09–2.27)**
	Half	21 (5.2)	187 (4.8)	1.22 (0.76–1.96)	1.11 (0.66–1.86)
	Three quarters	13 (3.2)	68 (1.8)	**2.08 (1.13–3.82)**	**2.10 (1.10–4.01)**
	Almost always	13 (3.2)	64 (1.6)	**2.21 (1.20–4.07)**	**2.00 (1.03–3.88)**
Severe ambient cooling	Never	231 (57.5)	2,723 (70.2)	Reference	Reference
	One tenth	81 (20.1)	740 (19.1)	1.29 (0.99–1.68)	**1.35 (1.01–1.82)**
	One quarter	41 (10.2)	198 (5.1)	**2.44 (1.70–3.51)**	**2.45 (1.65–3.64)**
	Half	22 (5.5)	119 (3.1)	**2.18 (1.36–3.50)**	**2.00 (1.18–3.40)**
	Three quarters	10 (2.5)	57 (1.5)	**2.07 (1.04–4.10)**	**2.09 (1.02–4.27)**
	Almost always	17 (4.2)	43 (1.1)	**4.66 (2.62–8.30)**	**4.02 (2.09–7.71)**

*OR* odds ratio, *NRS* numerical rating scale, *95% CI* ninety-five percent confidence interval

^a^Adjusted for age, gender, body mass index, current daily smoking, diabetes mellitus, joint disease, and hand-arm vibration exposure

Bold values are significant at the 0.05 level

### Relation between exposure variables

Occupational exposure to HAV for more than one tenth of the working time was reported by 333 (7.7%) subjects, while heavy manual handling to the same extent was reported by 1,319 (30.4%) subjects. There were statistically significant correlations between the three cold exposure variables and HAV, with correlation coefficients ranging from 0.44 to 0.54 (*p* < 0.001), as well as for the cold exposure variables and heavy manual handling, where the correlation coefficients ranged from 0.47 to 0.54 (*p* < 0.001). Analyses on interaction effects between cold and HAV exposure were performed, but limited by a low number of subjects in some exposure categories (Additional file 1). However, there were positive additive interaction effects between the cold exposure variables and heavy manual handling (Table 3). For workers exposed to severe ambient cooling for more than half of their working hours, in addition to performing heavy manual handling every day, the OR (95% CI) for reporting CTS symptoms was 7.25 (3.88–13.53), with a positive additive interaction effect (expressed as relative excess risk due to interaction) of 4.67. In this group, the three most common occupational groups were construction workers (*N* = 13; 14.9%), drivers of transport and construction vehicles (*N* = 11; 12.6%), and metal workers (*N* = 6; 6.9%).

**Table 3:** Interaction between occupational cold and biomechanical exposures in relation to symptoms of carpal tunnel syndrome

**Exposure variables**		**Carpal tunnel syndrome/Total**	**Simple analyses**	**Multiple analyses **^**a**^	**Relative excess risk due to interaction **^**b**^
Proportion of working hours		N	OR (95% CI)	OR (95% CI)	
Contact cooling	Heavy manual handling ^c^				
Never	Never	196/2,631	Reference	Reference	
Never	Every day	17/117	**2.13 (1.25–3.64)**	**2.15 (1.22–3.80)**	
Half the time or more	Never	5/31	2.56 (0.97–6.78)	1.61 (0.52–5.02)	
Half the time or more	Every day	25/90	**4.80 (2.96–7.79)**	**4.22 (2.18–8.16)**	1.46
Ambient cooling	Heavy manual handling ^c^				
Never	Never	178/2,324	Reference	Reference	
Never	Every day	11/89	1.72 (0.90–3.30)	1.42 (0.70–2.88)	
Half the time or more	Never	4/72	0.72 (0.26–2.00)	0.68 (0.24–1.94)	
Half the time or more	Every day	30/123	**3.92 (2.53–6.09)**	**3.45 (1.92–6.22)**	2.35
Severe ambient cooling	Heavy manual handling ^c^				
Never	Never	175/2,470	Reference	Reference	
Never	Every day	20/122	**2.59 (1.56–4.29)**	**2.64 (1.53–4.55)**	
Half the time or more	Never	6/60	1.49 (0.63–3.53)	0.94 (0.33–2.70)	
Half the time or more	Every day	28/90	**6.04 (3.76–9.71)**	**7.25 (3.88–13.53)**	4.67

^a^Adjusted for age, gender, body mass index, current daily smoking, diabetes mellitus, joint disease, and hand-arm vibration exposure

^b^Calculated based on odds ratios from multiple analyses, where a value > 0 indicate a positive additive interaction effect

^c^Lifting at least 15 kg per unit multiple times per day

Bold values are significant at the 0.05 level

## Discussion

This population-based study on working-age subjects indicated that self-reported occupational exposure to contact and ambient cooling was associated with symptoms of carpal tunnel syndrome, after adjusting for age, gender, body mass index, current daily smoking, diabetes mellitus, joint disease, and hand-arm vibration exposure. There were positive exposure–response patterns for time spent in different cooling conditions during work and reporting symptoms of carpal tunnel syndrome. There were also positive additive interaction effects between occupational cold exposure and heavy manual handling.

As concluded in a previous review, very few studies have investigated cold exposure as a risk factor for CTS [25]. To our knowledge, only one study has included contact cooling, and found an OR of 9.4 (95% CI 2.4–37.2) for CTS among workers exposed to both contact cooling and repetitive wrist movements with unexposed office workers as the reference group, compared to an OR of 2.2 (95% CI 0.2–21.2) for workers only exposed to repetitive wrist movements, after adjusting for age, gender, and length of employment [13]. That particular study was conducted among Taiwanese frozen food factory workers (*N* = 207), who were heavily exposed to contact cooling from handling of foodstuff between − 12 and − 15 °C, with reduced finger skin temperatures of around 26 to 28 °C, despite wearing gloves. In our study, the association between contact cooling and CTS symptoms was not as prominent (adjusted OR 3.20; 95% CI 1.62–6.33 among those with the most intense exposure), but only the duration and not the intensity of the exposure was inquired for. Since previous studies have shown that only roughly 4% of Swedish workers are employed in artificially refrigerated environments [26], and the mean monthly outdoor temperature during the study period ranged from − 6.6 to 3.5 °C [27], it is reasonable to assume that the temperature of the goods handled by the exposed subjects in our study could have been substantially less cold, which would explain the smaller effect size. Also, the study on frozen food factory workers was conducted in a subtropical climate, where long-term cold adaptation mechanisms are likely less pronounced than in northern Sweden [28], which could also serve to explain the difference in effect size. Apart from effects of contact cooling, a previous study with case–control design (*N* = 229) concluded that working in cold environments was also associated with surgically treated CTS (OR 3.52; 95% CI 1.08–11.47), as determined by both clinical symptoms and neurophysiological studies, after adjusting for repetitive wrist movements, BMI, and educational level [5]. That study was performed in Israel, which has a subtropical climate, and it was not specified if cold exposure occurred in artificially cooled indoor environments or outdoors. Their findings were in line with our results, where we found associations to both cold environments in general (adjusted OR 2.00; 95% CI 1.03–3.88) and more extreme conditions with cold, moisture, and wind (adjusted OR 4.02; 95% CI 2.09–7.71). Finally, our finding of an association between manifest cold injury and CTS symptoms is also supported by previous literature [15], but the time relation has not been concluded on (i.e. if cold injury precedes CTS symptoms, or if the opposite is true).

The pathophysiological mechanisms behind an increased occurrence of CTS in relation to cold exposure are not entirely understood. It has been shown that long-standing compression of the median nerve at the wrist can impair both arterial and venous intraneural blood supply, and that contact cooling can further attenuate such decreased perfusion, when assessed by Doppler ultrasonography in a case–control study (*N* = 100) [29]. However, short-term cooling of the hand in asymptomatic individuals (*N* = 10) did not affect ultrasonographic measures of median nerve diameter or transverse carpal ligament elasticity, indicating that the mechanical properties (i.e. swelling and rigidity) of the structures are not likely culprits [30]. In an experimental study (*N* = 46), subjects with CTS were found to have a more pronounced distal sensory latency after immersion in ice-cold water than healthy controls, indicating an increased neural sensitivity to cold exposure among those with compression neuropathy [20]. Apart from vascular effects, cooling also has an immediate effect on the neural transmission in itself, by affecting voltage-gated ion channels along the axon, inducing a slower conduction velocity [16].

Since previous literature reviews have reported that repetitive wrist movements and forceful gripping increase the risk of CTS [6, 9], it would have been valuable to include such parameters in our models. To account for biomechanical exposure, we used an exposure variable for heavy manual handling. However, this variable was not specific with regards to grip force or wrist movements, and was also strongly correlated to cold exposure, which made it unsuitable as a covariate in the multiple models. In addition, when included empirically, it reduced the goodness-of-fit of the models (data not shown). However, heavy manual handling was included in separate interaction analyses, where there were positive additive interaction effects (Table 3), and this seems very plausible from a mechanistic standpoint. For example, a cold store worker can be exposed to both contact cooling when handling frozen goods, but also heavy manual handling. However, in such an instance, it is not realistic to separate these exposures by stratification or confounder adjustment. We believe that heavy manual handling better reflects the use of forceful grip than repetitive wrist movements, but the validity in the exposure assessment can still be questioned. We have found only one previous study examining interaction effects, and they concluded on a statistically significant positive interaction between contact cooling of the hands and repetitive wrist movements in relation to CTS among frozen food workers [13]. However, it should be stated that in our study, subjects with both high cold exposure and heavy manual handling were mostly employed in the construction sector, and not in the frozen food industry. Exposure to HAV is another important occupational risk factor for CTS. However, most subjects in our study were unexposed, and interaction analyses were hampered by insufficient statistical power, but still suggestive of a positive additive interaction effect (Additional file 1). Furthermore, we adjusted our multiple models for HAV exposure but this did not have any major effect on the associations between cold exposure and CTS symptoms. In other settings, HAV exposure might play a larger role. To conclude, we believe that further studies are needed to evaluate the effect sizes of occupational contact and ambient cooling in relation to repetitive wrist movements, forceful gripping, and HAV in greater detail.

There are reasons to believe that gender plays an important role in the development of CTS. To begin with, CTS due non-occupational factors are more common among women [2], being related to both body composition and pregnancy [3]. In contrast, several established occupational risk factors for CTS are more common among men [6]. When building our multiple logistic regression models, we used gender as a covariate, but this had little impact on the effect sizes (Table 2). In addition, we also performed gender-stratified analyses, which mainly showed associations between cold exposure and CTS symptoms among males (Additional file 1). However, there were very few female cases in the higher cold exposure categories, which raises concern about statistical power, and the potential for type 2 error. We therefore encourage further studies on this topic, preferably with a larger sample size of cold-exposed women, that could for instance be recruited from large frozen food factories or childcare centers.

According to our survey, performed in northern Sweden, numbness or tingling in the fingers innervated by the median nerve was reported by roughly 13%. This was very similar to a previous population-based study performed in southern Sweden (*N* = 2,466), where pain, numbness or tingling in the median nerve distribution of the hands was reported by 14% [4]. However, among symptomatic subjects in the latter study, only 4% were considered to have clinically certain CTS after examination by a physician, and merely 3% fulfilled both clinical and neurophysiological criteria. Thus, the occurrence rate acquired in a postal survey may very well be an overestimation from a clinical viewpoint. The gold standard for diagnosing CTS is currently considered to be nerve conduction studies [3]. Different case definitions for CTS in epidemiological studies can result in a range of prevalence estimates and a risk of misclassification [31]. In our study, we tried to increase the specificity by also requiring nocturnal symptoms for our case definition, as has been previously suggested [3], and this reduced the prevalence to about 9%. Although still likely an exaggeration of the true prevalence, it was not considered feasible to invite all participants for clinical or neurophysiological examination due to the large sample size. Also, having a slightly too inclusive case definition would likely attenuate statistical associations with occupational factors. We therefore believe that our results are valid, and associations not overestimated.

An important limitation in our study was the fact that the exposure data was subjectively reported and only quantified by exposure duration, but not in terms of intensity. Future studies would benefit from performing objective measurements of ambient and surface temperatures, and assessing the validity of self-reported exposure in relation to actual field measurements. It would also be valuable to collect data on the use of personal protective equipment, such as warm gloves. Data on occupation (categorized using the major and sub-major ISCO groups) were not detailed enough to enable the use of multi-level job-exposure matrices. We lacked valid exposure data on important occupational risk factors such as repetitive wrist movements and forceful gripping. The mainly cross-sectional design meant that the time-relation between exposure and outcome could not be established. However, in a complementary longitudinal analysis, there was also an association between occupational cold exposure in 2015 and CTS symptoms in 2021, indicating that the main findings were unlikely to be explained by reverse causation. Further, the low response rate (44%) could have introduced a sampling bias and reduced the generalizability of the results. If asymptomatic subjects were less prone to answer the survey, this would have exaggerated prevalence estimates of CTS symptoms. There is also a risk of systematic bias in exposure assessments, which potentially could have both augmented and attenuated statistical associations. Thus, the results in our study should mainly be considered to be hypothesis-generating, and subject for future confirmative studies of other design. However, when comparing our results to the previous literature, although scarce, we believe that our results are well in line with was has been found by other authors. Also, to our knowledge, this is by far the largest epidemiological study conducted on the topic to date, and it collected ample background data on study participants to enable thorough adjustment of regression analyses, which we believe increase the robustness of the conclusions in this study.

Swedish workplace regulation cover exposure to HAV, hand-intensive work tasks, and contact cooling, but not in any detailed manner the effects of ambient cooling. In the provisions on workplace design, it is mandated that that the employer should arrange the work to prevent cooling of the hands, and provide personal protective equipment to protect from contact cooling [32]. To fulfill the requirements, preventive measures should be undertaken, preferably guided by the ISO standard for contact cooling (ISO 13732–3:2005) and medical surveillance of cold-exposed workers (ISO 15743:2008). In light of the findings in our study, we believe that ambient cold exposure during work could also be more closely regulated.

## Conclusions

Self-reported occupational exposure to contact and ambient cooling was associated with self-reported symptoms suggestive of carpal tunnel syndrome. There were statistically significant positive exposure–response patterns for time spent exposed to contact and ambient cooling at work in relation to reporting symptoms of carpal tunnel syndrome. Positive additive interaction effects between cold exposure and heavy manual handling were also found. Since there was important potential uncontrolled confounding regarding repetitive wrist movements and forceful gripping, the results need to be confirmed by other studies, preferably with longitudinal design and more detailed exposure assessment.

## Supplementary Information

**Additional file 1. **Additional gender-stratified analyses and interaction analyses for cold and hand-arm vibration exposure.

## Data Availability

The datasets used and analyzed during the current study are available from the corresponding author on reasonable request.

## Abbreviations

BMI: Body mass index
CTS: Carpal tunnel syndrome
HAV: Hand-arm vibration
ISCO: International standard classification of occupations
ISO: International organization for standardization
IQR: Interquartile range
NRS: Numerical rating scale
OR: Odds ratio
RERI: Relative excess risk due to interaction

## References

[1] Newington L, Harris EC, Walker-Bone K (2015). Carpal tunnel syndrome and work. Best Pract Res Clin Rheumatol.

[2] de Krom MC, Knipschild PG, Kester AD, Thijs CT, Boekkooi PF, Spaans F (1992). Carpal tunnel syndrome: prevalence in the general population. J Clin Epidemiol.

[3] Alfonso C, Jann S, Massa R, Torreggiani A (2010). Diagnosis, treatment and follow-up of the carpal tunnel syndrome: a review. Neurol Sci.

[4] Atroshi I, Gummesson C, Johnsson R, Ornstein E, Ranstam J, Rosen I (1999). Prevalence of carpal tunnel syndrome in a general population. JAMA.

[5] Yagev Y, Gringolds M, Karakis I, Carel RS (2007). Carpal tunnel syndrome: under-recognition of occupational risk factors by clinicians. Ind Health.

[6] Palmer KT (2011). Carpal tunnel syndrome: the role of occupational factors. Best Pract Res Clin Rheumatol.

[7] Tadjerbashi K, Åkesson A, Atroshi I (2019). Incidence of referred carpal tunnel syndrome and carpal tunnel release surgery in the general population: Increase over time and regional variations. J Orthop Surg (Hong Kong).

[8] Geoghegan JM, Clark DI, Bainbridge LC, Smith C, Hubbard R (2004). Risk factors in carpal tunnel syndrome. J Hand Surg Br.

[9] Barcenilla A, March LM, Chen JS, Sambrook PN (2012). Carpal tunnel syndrome and its relationship to occupation: a meta-analysis. Rheumatology (Oxford).

[10] Vihlborg P, Pettersson H, Makdoumi K, Wikström S, Bryngelsson IL, Selander J (2022). Carpal Tunnel Syndrome and Hand-Arm Vibration A Swedish National Registry Case-Control Study. J Occup Environ Med.

[11] International Organization for Standardization (2008). ISO 15743:2008 - Ergonomics of the thermal environment - Cold workplaces - Risk assessment and management.

[12] Mäkinen TM, Hassi J (2009). Health problems in cold work. Ind Health.

[13] Chiang HC, Chen SS, Yu HS, Ko YC (1990). The occurrence of carpal tunnel syndrome in frozen food factory employees. Gaoxiong Yi Xue Ke Xue Za Zhi.

[14] Scalco RS, Pietroski F, Celli LF, Gomes I, Becker J (2013). Seasonal variation in prevalence of carpal tunnel syndrome. Muscle Nerve.

[15] Golant A, Nord RM, Paksima N, Posner MA (2008). Cold exposure injuries to the extremities. J Am Acad Orthop Surg.

[16] Rutkove SB (2001). Effects of temperature on neuromuscular electrophysiology. Muscle Nerve.

[17] Jia JP, Pollock M (1997). The pathogenesis of non-freezing cold nerve injury - Observations in the rat. Brain.

[18] Hutchison RL (2014). Frostbite of the Hand. J Hand Surg-Am.

[19] Vale TA, Symmonds M, Polydefkis M, Byrnes K, Rice ASC, Themistocleous AC (2017). Chronic non-freezing cold injury results in neuropathic pain due to a sensory neuropathy. Brain.

[20] de Araujo RGM, Kouyoumdjian JA (2007). Cooling modifies mixed median and ulnar palmar studies in carpal tunnel syndrome. Arq Neuro-Psiquiat.

[21] Stjernbrandt A, Björ B, Andersson M, Burström L, Liljelind I, Nilsson T (2017). Neurovascular hand symptoms in relation to cold exposure in northern Sweden: a population-based study. Int Arch Occup Environ Health.

[22] International Labour Organization (2012). International Standard Classification of Occupations (ISCO-08).

[23] World Health Organization (1995). Physical status: the use and interpretation of anthropometry.

[24] VanderWeele TJ, Knol MJ (2014). A Tutorial on Interaction Epidemiologic Methods.

[25] Pienimäki T (2002). Cold exposure and musculoskeletal disorders and diseases. A review Int J Circumpolar Health.

[26] Stjernbrandt A. Cold exposure and health: a study on neurological and vascular hand symptoms in northern Sweden [Thesis]. 2021.

[27] The Swedish Meteorological and Hydrological Institute (SMHI). National Weather Data Report. The Swedish Meteorological and Hydrological Institute; 2021.

[28] Mäkinen TM (2007). Human cold exposure, adaptation, and performance in high latitude environments. Am J Hum Biol.

[29] Chang YW, Chen CJ, Wang YW, Chiu V, Lin SK, Horng YS (2021). Influence of temperature on sonographic images of the median nerve for the diagnosis of carpal tunnel syndrome: a case control study. Bmc Medical Imaging..

[30] Laymon M, Petrofsky J, McKivigan J, Lee H, Yim J (2015). Effect of heat, cold, and pressure on the transverse carpal ligament and median nerve: a pilot study. Med Sci Monitor..

[31] Descatha A, Dale AM, Franzblau A, Coomes J, Evanoff B (2011). Comparison of research case definitions for carpal tunnel syndrome. Scand J Work Environ Health.

[32] Swedish Work Environment Authority (2020). AFS 2020:1 – Workplace design.

