# Supplementary material for: Occupational cold exposure and symptoms of carpal tunnel syndrome – a population-based study
Source: BMC Musculoskelet Disord. 2022 Jun 21;23:596. doi: 10.1186/s12891-022-05555-8 (PMC9210706; doi:10.1186/s12891-022-05555-8)
Supplement: Supplementary file 1 — Additional file 1. Additional gender-stratified analyses and interaction analyses for cold and hand-arm vibration exposure. [file 12891_2022_5555_MOESM1_ESM.pdf]

Additional file 1

Title of paper: Occupational cold exposure and symptoms of carpal tunnel syndrome – a population-based study

Authors: Albin Stjernbrandt, Per Vihlborg, Viktoria Wahlström, Jens Wahlström, Charlotte Lewis

Journal: BMC Musculoskeletal Disorders

Table 4 Gender-stratified symptom-based case definition for carpal tunnel syndrome

**Variable**	**Male** N (%)	**Female** N (%)
Having tingling or numbness in the thumb, index and middle finger	291 (12.8%)	367 (13.8%)
Having nocturnal numbness in the hands	328 (14.4%)	560 (21.0%)
Positive answers to both items above (case definition)	180 (8.0%)	273 (10.3%)

Table 5 Gender-stratified logistic regression for occupational cold exposure in relation to reporting symptoms of carpal tunnel syndrome

**Gender**	**Exposure variable**	**Exposure level** (Proportion of working hours)	**Carpal tunnel syndrome** N (%)	**References** N (%)	**Simple analyses** Crude OR (95% CI)	**Multiple analyses** [a] Adjusted OR (95% CI)
Male	Contact cooling	Never	65 (40.6)	1,151 (61.2)	Reference	Reference
		One tenth	43 (26.9)	440 (23.4)	**1.73 (1.16–2.58)**	1.28 (0.81–2.03)
		One quarter	20 (12.5)	140 (7.4)	**2.53 (1.49–4.30)**	1.66 (0.90–3.07)
		Half	10 (6.3)	67 (3.6)	**2.64 (1.30–5.38)**	1.89 (0.86–4.14)
		Three quarters	12 (7.5)	51 (2.7)	**4.17 (2.12–8.20)**	**2.63 (1.22–5.64)**
		Almost always	10 (6.3)	31 (1.6)	**5.71 (2.68–12.16)**	**3.86 (1.69–8.82)**
	Ambient cooling	Never	59 (36.9)	961 (51.3)	Reference	Reference
		One tenth	42 (26.3)	471 (25.1)	1.45 (0.96–2.19)	1.12 (0.71–1.77)
		One quarter	26 (16.3)	205 (10.9)	**2.07 (1.27–3.36)**	1.44 (0.83–2.49)
		Half	11 (6.9)	131 (7.0)	1.37 (0.70–2.67)	0.94 (0.45–1.94)
		Three quarters	12 (7.5)	51 (2.7)	**3.83 (1.94–7.58)**	**2.70 (1.29–5.69)**
		Almost always	10 (6.3)	55 (2.9)	**2.96 (1.44–6.10)**	1.90 (0.86–4.20)
	Severe ambient cooling	Never	59 (37.1)	1,092 (58.2)	Reference	Reference
		One tenth	44 (27.7)	493 (26.3)	**1.65 (1.10–2.48)**	1.39 (0.89–2.17)
		One quarter	23 (14.5)	137 (7.3)	**3.11 (1.86–5.19)**	**2.23 (1.26–3.96)**
		Half	15 (9.4)	83 (4.4)	**3.35 (1.82–6.15)**	**2.34 (1.19–4.63)**
		Three quarters	8 (5.0)	43 (2.3)	**3.44 (1.55–7.66)**	**2.53 (1.08–5.97)**
		Almost always	10 (6.3)	29 (1.5)	**6.38 (2.97–13.72)**	**4.55 (1.99–10.39)**
Female	Contact cooling	Never	189 (78.4)	1,718 (85.8)	Reference	Reference
		One tenth	31 (12.9)	205 (10.2)	1.38 (0.92–2.06)	1.13 (0.73–1.76)
		One quarter	8 (3.3)	42 (2.1)	1.73 (0.80–3.74)	1.60 (0.71–3.60)
		Half	6 (2.5)	15 (0.7)	**3.64 (1.39–9.48)**	2.30 (0.78–6.82)
		Three quarters	3 (1.2)	11 (0.5)	2.48 (0.69–8.97)	2.09 (0.53–8.23)
		Almost always	4 (1.7)	11 (0.5)	**3.31 (1.04–10.48)**	2.19 (0.57–8.37)
	Ambient cooling	Never	168 (68.9)	1,509 (75.1)	Reference	Reference
		One tenth	40 (16.4)	295 (14.7)	1.22 (0.84–1.76)	1.14 (0.76–1.69)
		One quarter	22 (9.0)	124 (6.2)	1.59 (0.99–2.58)	**1.78 (1.07–2.96)**
		Half	10 (4.1)	56 (2.8)	1.60 (0.80–3.20)	1.44 (0.68–3.08)
		Three quarters	1 (0.4)	17 (0.8)	0.53 (0.07–4.00)	0.55 (0.07–4.25)
		Almost always	3 (1.2)	9 (0.4)	2.99 (0.80–11.17)	2.25 (0.57–8.93)
	Severe ambient cooling	Never	172 (70.8)	1,631 (81.4)	Reference	Reference
		One tenth	37 (15.2)	247 (12.3)	1.42 (0.97–2.08)	1.35 (0.90–2.03)
		One quarter	18 (7.4)	61 (3.0)	**2.80 (1.62–4.84)**	**2.98 (1.68–5.26)**
		Half	7 (2.9)	36 (1.8)	1.84 (0.81–4.21)	1.58 (0.63–3.96)
		Three quarters	2 (0.8)	14 (0.7)	1.36 (0.31–6.01)	1.33 (0.29–6.02)
		Almost always	7 (2.9)	14 (0.7)	**4.74 (1.89–11.91)**	**3.27 (1.05–10.16)**

OR odds ratio, *NRS* numerical rating scale, *95% CI* ninety-five percent confidence interval

[a] Adjusted for age, gender, body mass index, current daily smoking, diabetes mellitus, joint disease, and hand-arm vibration exposure

Bold values are significant at the 0.05 level

Table 6 Interaction between occupational cold and hand-arm vibration exposures in relation to symptoms of carpal tunnel syndrome

**Exposure variables**<br>Proportion of working hours		**Carpal tunnel syndrome/Total**<br>N	**Simple analyses**<br>OR (95% CI)	**Multiple analyses** [a]<br>OR (95% CI)	**Relative excess risk due to interaction** [b]
Contact cooling	Hand-arm vibration				
Never	Never	230/2,970	Reference	Reference	
Never	Every day	0/10	1.0 (-)	1.0 (-)	
Half the time or more	Never	8/53	2.09 (0.97–4.48)	1.51 (0.64–3.54)	
Half the time or more	Every day	7/27	**4.33 (1.80–10.40)**	**4.93 (1.76–13.83)**	3.42
Ambient cooling	Hand-arm vibration				
Never	Never	198/2,559	Reference	Reference	
Never	Every day	1/13	0.98 (0.13–7.56)	1.35 (0.16–10.99)	
Half the time or more	Never	11/147	0.95 (0.51–1.78)	0.86 (0.43–1.69)	
Half the time or more	Every day	5/24	**3.26 (1.20–8.87)**	**3.90 (1.29–11.82)**	2.69
Severe ambient cooling	Hand-arm vibration				
Never	Never	191/2,610	Reference	Reference	
Never	Every day	1/9	1.56 (0.19–12.52)	2.13 (0.22–20.78)	
Half the time or more	Never	21/172	**1.77 (1.09–2.86)**	1.65 (0.96–2.84)	
Half the time or more	Every day	3/9	**6.23 (1.55–25.12)**	**9.36 (2.11–41.47)**	6.58

[a] Adjusted for age, gender, body mass index, current daily smoking, diabetes mellitus, joint disease, and heavy manual handling

[b] Calculated based on odds ratios from multiple analyses, where a value >0 indicate a positive additive interaction effect

Bold values are significant at the 0.05 level
